# Two new factors modulating *Bacillus subtilis* spore resistance and germination

**DOI:** 10.1128/msphere.00741-25

**Published:** 2026-03-30

**Authors:** William J. Bannon, Dejalee Padilla, Dhruv Suryadevara, Faith Ye, George Korza, James Wicander, Peter Setlow

**Affiliations:** 1 Department of Molecular Biology and Biophysics, UConn Health 705913, Farmington, Connecticut, USA; The University of Iowa, Iowa City, Iowa, USA

**Keywords:** sporulene, bacillithiol, *Bacillus subtilis*, spores, spore UV resistance, spore chemical resistance, spore germination, spore pigmentation, spore inner membrane

## Abstract

New effectors of spore properties have been identified in two *Bacillus subtilis* strains—bacillithiol, the major low-molecular-weight thiol in *B. subtilis*, and sporulene, a multi-ring compound synthesized from curcumene in spores’ outer layers. The absence of either bacillithiol or sporulene caused faster germination of spores of one wild-type *B. subtilis* strain, PS832, with two germinant receptors (GR)-dependent germinants, L-valine for the GerA GR, and the AGFK mixture for the cooperative action of the GerB and GerK GRs, as well as the GR-independent germinant dodecylamine. However, these effects on PS832 spore germination were due to the presence of the antibiotic marker used to replace the genes for bacillithiol or sporulene synthesis. The absence of bacillithiol also caused reduced spore resistance to wet heat but not to hydrogen peroxide or UV radiation, while sporulenes’ absence had no effect on spore wet heat resistance, but reduced spore resistance to hydrogen peroxide as found previously (T. Bosak, R. M. Losick, and A. Pearson, Proc Natl Acad Sci USA 105:6725-6729, 2008, https://doi.org/10.1073/pnas.0800199105) and to UV radiation, but the presence of the antibiotic markers in these mutant strains was not responsible for these effects. Notably, spores lacking sporulene were much whiter than wild-type (wt) spores, suggesting that sporulene and its precursor curcumene contribute to spore pigmentation that can absorb UV, thus reducing spores’ UV resistance. Analyses of reasons for the effects of bacillithiol’s or sporulene’s absence on spore resistance, specifically core water content and/or altered spore inner membrane (IM) permeability, found no major differences from values in wt spores. However, other analyses showed that sporulene’s absence led to slight changes in spore IM fluidity.

**IMPORTANCE:**

The work in this paper has identified two new factors, bacillithiol and sporulene, as modulators of the resistance and germination of spores of two *Bacillus subtilis* strains, and by extension, probably spores of other Bacillota. Since spores of some species can give rise to cells that can cause food spoilage and/or disease, new knowledge about spore resistance and germination could have applied utility.

## INTRODUCTION

The metabolic dormancy, resistance, and return to life in germination (1–3) of spores of Bacillota species are of basic interest because of how these spore properties are achieved. In addition, spores of many of these species are vectors of food spoilage and disease (3), and thus, their dormancy, resistance, and germination are of applied interest. Previous work (2) has shown that multiple factors contribute to spore resistance, including (i) spore core’s water content, as low as 25% of wet wt; (ii) high core levels of the 1:1 conjugate of Ca^2+^ with 2,6-dicarboxypicolinic acid (CaDPA; ~20% of spore core dry wt); (iii) spore-specific DNA binding proteins; (iv) the inner membrane (IM) around the spore core that has low permeability and lipid mobility; (v) multiple IM proteins that modulate IM permeability and lipid immobility; and (vi) large amounts of protein in spores’ outer layers that detoxify hazardous chemicals. Many of these factors, particularly IM fluidity, also modulate spore germination (4–8). Notably, despite years of research on spore resistance and germination, novel factors continue to be discovered, such as the recent identification of novel IM proteins that affect both spore resistance and germination (4, 5, 8). Consequently, it is possible that additional factors affecting *Bacillus* spore resistance and germination remain to be discovered.

The current work focuses on the following two minimally studied factors for their effects on *B. subtilis* spore germination and resistance: (i) bacillithiol and (ii) sporulene (9–14). Bacillithiol is the principal low-molecular-weight thiol in *Bacillus* species, which lacks glutathione and plays a significant role in growing cells’ resistance to oxidative stress (13, 14). However, bacillithiol’s effects on spore resistance and germination have not been studied. Sporulene is a very hydrophobic terpene-based lipid likely in the outer layers of spores of *B. subtilis* and probably the spores of many Bacillota, and it is synthesized in the mother cell compartment of the sporulating cell (9–11, 15). Sporulene and its precursor curcumene contribute to spores’ hydrogen peroxide resistance (9, 12), but contributions to other spore properties have not been examined. Given the unexplored aspects of these two spore constituents, we have examined the effects of loss of bacillithiol or sporulene on *B. subtilis* spore IM permeability, fluidity, germination, and resistance to UV light, wet heat, hydrogen peroxide, and formaldehyde, as well as spore core water content.

## MATERIALS AND METHODS

### *B. subtilis* strains and spore preparation and purification

The *B. subtilis* strains used in this work are isogenic with either PS832 (wild-type [wt]), a laboratory 168 isolate, or PY79 (also wt) and are listed in Table 1. New strains were generated by transformation of PS832 or PY79 with chromosomal DNA from antibiotic-resistant deletion replacement mutant strains (15) from the *Bacillus* Genetic Stock Center to generate strains with an antibiotic resistance gene replacing a gene essential for synthesis of sporulene (*sqhC*), strains PS4559 and PS4597, or bacillithiol (*bshC*), strain PS4560 (6, 13). Strains PS4561 and PS4562 were derived from strains PS4559 and PS4560, respectively, by removing the antibiotic resistance genes using the Cre recombinase as described (16). PCR and genome sequencing were carried out on new strains to assure they had the correct genotypes. Spores of strains were prepared by growth at 37°C on 2× SG nutrient medium plates for ~3 days at 37°C, and the spores were harvested and extensively purified, including a final centrifugation in a 50% Nycodenz solution in which dormant spores’ pellet while vegetative and sporulating cells, germinated spores, and debris float (17, 18), and they can be discarded. All spores used were >99% free of growing or sporulating cells, germinated spores, and visible debris, and were stored at 4°C at an optical density at 600 nm (OD_600_) of 10–20, protected from light, and used within 4 months.

**TABLE 1:** *B. subtilis* strains used in this work^*a*^

Strain	Genotype	Antibiotic marker	Source
PS832^*b*^	wt	None	Laboratory strain
PS4559^*b*^	Δ*sqhC*	Km^r^	This work
PS4561	Δ*sqhC*	None	This work
PS4560^*b*^	Δ*bshC*	Em^r^	This work
PS4562^*b*^	Δ*bshC*	None	This work
PY79^*c*^	wt	None	Laboratory strain
PS4567^*c*^	Δ*sqhC*	Km^r^	This work

^*a*^ Strains were made in this work as described in Mataerials and Methods by replacing coding regions of either the *sqhC* or *bshC* genes with kanamycin (Km^r^) or erythromycin (Em^r^) resistance markers, respectively, and in some cases, antibiotic resistance genes were removed using the Cre recombinase (15).

^*b*^ PS832 background.

^*c*^ PY79 background.

### Spore resistance measurements

Determination of the resistance of spores at an OD_600_ of ~1 (~10^8^ spores/mL) in water at 93°C, UV light at 254 nm, 11% hydrogen peroxide (H_2_O_2_), or 2.5% formaldehyde (HCHO) was carried out in duplicate as described previously (5, 6, 19), with duplicate determinations in each experiment; data points at the same times were averaged, and statistical significances were determined as described below.

### Spore germination

Spores were germinated with two germinant receptors (GR)-dependent germinants following heat activation at 70°C (1, 20), 30 min for L-valine germination, and 90 min for germination with the AGFK mixture, with subsequent cooling on ice. Germination was assessed by monitoring release of spores’ CaDPA in a fluorometric plate reader (21) at 37°C with spores at an OD_600_ of 0.5 (~5 × 10^7^ spores/mL) in 200 µL of 25 mM K-Hepes buffer (pH 7.4) with 50 µM TbCl_3_ and either 10 mM L-valine or a mixture of 10 mM each of L-asparagine, D-glucose, D-fructose, and KCl (AGFK). Germination was routinely followed for 3 h with duplicate measurements of Tb-DPA fluorescence every 5 min, and duplicate values were averaged. For germination with the GR-independent germinant dodecylamine, there was no heat activation, and spores at an OD_600_ of 1 were incubated at 45°C in 2 mL of 1 mM dodecylamine plus 25 mM K-Hepes buffer (pH 7.4). At various times, 100 µL aliquots were added to 100 µL of 100 µM TbCl_3_ in 25 mM K-Hepes buffer (pH 7.4); Tb-DPA fluorescence was measured in duplicate, and values were averaged.

### Statistical analyses

Statistical analyses were performed using GraphPad Prism 10 (GraphPad Software, San Diego, CA). For experiments comparing two groups, unpaired Student’s *t*-tests were used. For experiments comparing three or more groups, one-way ANOVA was performed first; when ANOVA indicated significant differences (*P* < 0.05), pairwise unpaired *t*-tests were conducted. For data with large dynamic ranges (colony-forming units, relative fluorescence units), log transformation was applied prior to statistical analysis. For percent viable data, no transformation was applied. Statistical significance was set at *P* < 0.05, and significant differences are indicated by asterisks in figures. All experiments included *n* = 2 biological replicates per group.

### Effects of the absence of sporulene or bacillithiol on spores’ IM permeability, fluidity, pigmentation, and core water content

The IM properties of spores of intact wt or PS4559 (no sporulene) or PS4560 (no bacillithiol) were measured in two ways: (i) measuring wt, sporulene-less and bacillithiol-less spores’ IM permeability by monitoring rates of uptake of ^14^C-methylamine (6) and (ii) by the fluorescence characteristics of well-purified wt, bacillithiol-less and sporulene-less spores labeled with 250 µM Laurdan on sporulation plates, and general polarization (GP) values reflecting IM fluidity were obtained as described (8, 22, 23). To assess spore pigmentation, 2.5 mL spores at an OD_600_ of ~25 that had been purified, including a final centrifugation through Nycodenz in which debris, germinated spores, and growing cells float and can be discarded (18), were pelleted, and the pellets were photographed. Core water content, a major contributor to spore resistance properties, was measured by centrifugation of intact mutant and wt spores to equilibrium in a Nycodenz density gradient and comparing banding positions, all as described (24, 25).

## RESULTS

### The absence of bacillithiol or sporulene had major effects on some spore resistance properties

**Fig 1:**
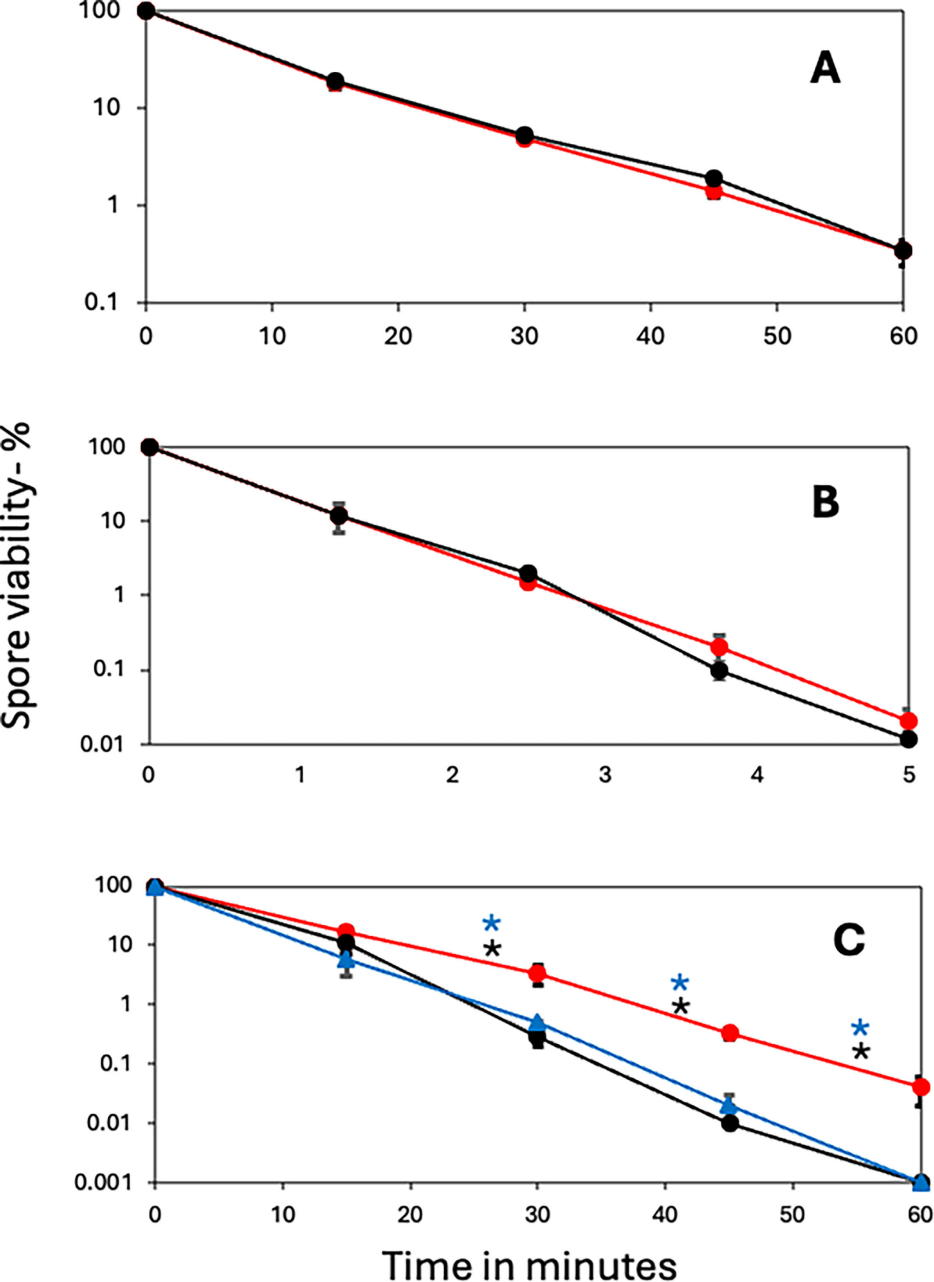
Resistance of PS832 spores with and without bacillithiol to various agents. Spores of strains PS832 (wt) and PS4560 (no bacillithiol, resistant to erythromycin) were (**A**) incubated with hydrogen peroxide and (**B**) exposed to UV radiation, and (**C**) spores of strains PS832, PS4560, and PS4562 (PS4560 with the antibiotic resistance marker removed) were exposed to wet heat at 93°C. Spore viability was determined as described in Materials and Methods. All values are averages of the results from duplicate determinations in at least two independent experiments. The symbols used are as follows: red circle and line, PS832 (wt); black circle and line, PS4560; and blue triangle and line, PS4562. Statistical significance was determined by one-way ANOVA (comparing PS832 wt, PS4560, and PS4562), followed by pairwise unpaired *t*-tests comparing each bacillithiol-less strain (PS4560 and PS4562) to wild-type PS832, and comparing PS4560 to PS4562, at each time point. Black and blue asterisks indicate significant differences (*P* < 0.05) for specific pairwise comparisons between PS832 and PS4560 and between PS832 and PS4562 at the indicated time points. There was no significant difference (*P* > 0.05) between PS4560 and PS4562.

**Fig 2:**
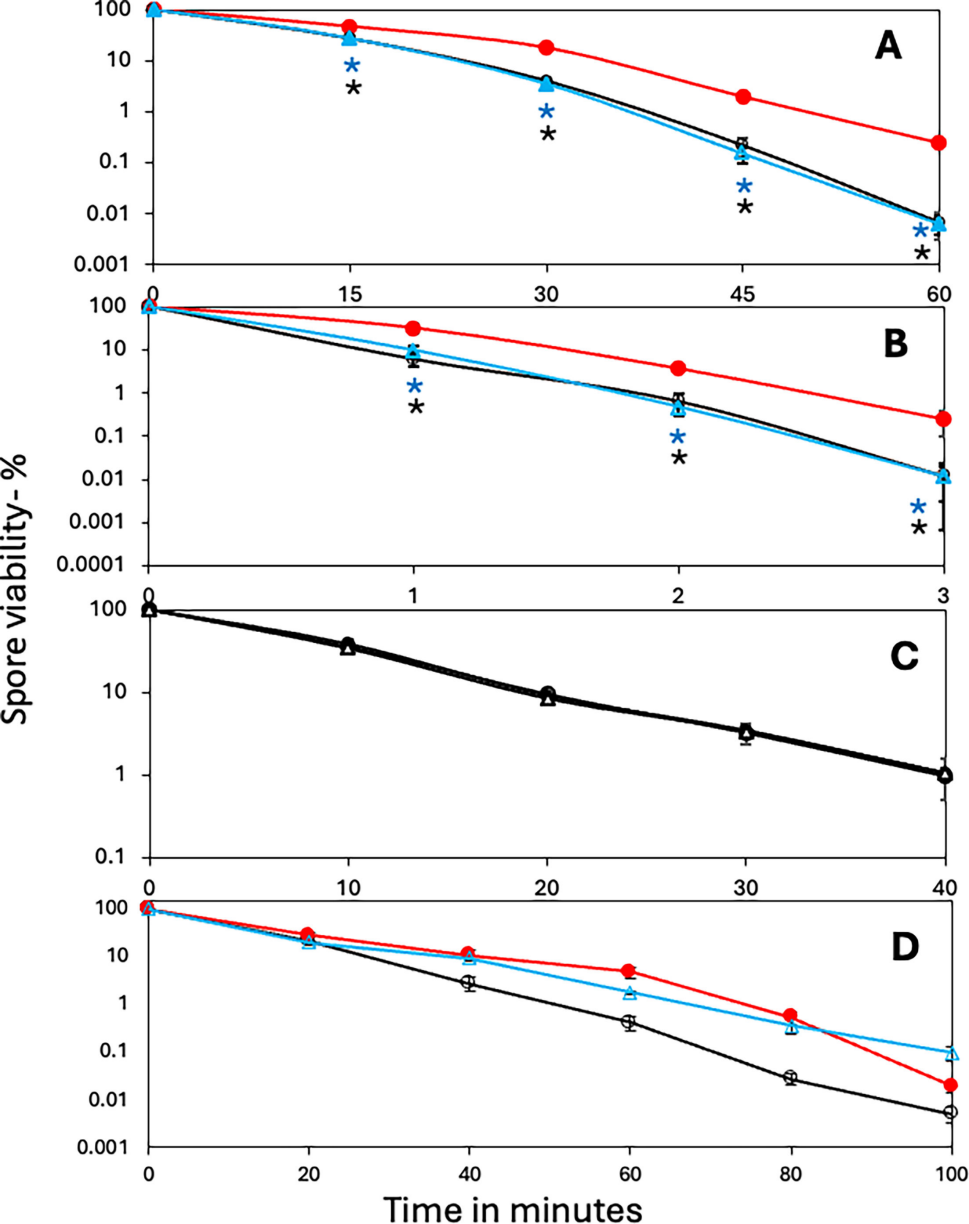
Resistance of spores of PS832 strains with or without sporulene to various agents. Spores of PS832 (wt), PS4559 (no sporulene), and PS4561 (PS4559 with the antibiotic resistance marker removed) were treated with (**A**) H_2_O_2_, (**B**) UV light, (**C**) wet heat at 93°C, or (**D**) HCHO, and spore viability was determined as described in Materials and Methods. All values and error bars (standard deviations) are averages from duplicate determinations in at least two independent experiments. The symbols used are as follows: red-filled circle and line, PS832 (wt); black open circle and line, PS4559 (sporulene-less); and blue triangle and line, PS4561, sporulene-less and with the kanamycin resistance gene removed. Statistical significance was determined by one-way ANOVA (comparing PS832 wt, PS4559, and PS4561), followed by unpaired *t*-tests comparing each sporulene-less strain (PS4559 and PS4561) to wt PS832 and comparing PS4559 to PS4561 at each time point. Black and blue asterisks indicate significant differences (*P* < 0.05) for specific pairwise comparisons between PS832 and PS4559 and between PS832 and PS4561 in regions shown by lines. There was no significant difference (*P* > 0.05) between PS4559 and PS4561.

Previous work has shown that the absence of bacillithiol from growing cells results in lower resistance to oxidizing agents or oxidative stress (13, 14), although effects of this thiol on spore resistance have not been studied. However, PS832 spore resistance to hydrogen peroxide or UV light was affected minimally, if at all, by bacillithiol’s absence (Fig. 1A and B). In contrast, spores of strain PS4560 lacking bacillithiol were significantly less wet heat resistant than wt spores (Fig. 1C). Importantly (see below), spores of strain PS4562 that lack bacillithiol and the antibiotic resistance marker in strain PS4560 exhibited the same lower wet heat resistance as that of PS4560 spores (Fig. 1C). The absence of bacillithiol also led to PS832 spores’ lower formaldehyde resistance, but removal of the antibiotic marker from the bacillithiol mutant eliminated the formaldehyde sensitivity (data not shown), as was also the case with spores carrying the sporulene deletion and having lost the antibiotic marker (Fig. 2D), indicating that the absence of sporulene alone had no effect on spore resistance to formaldehyde.

**Fig 3:**
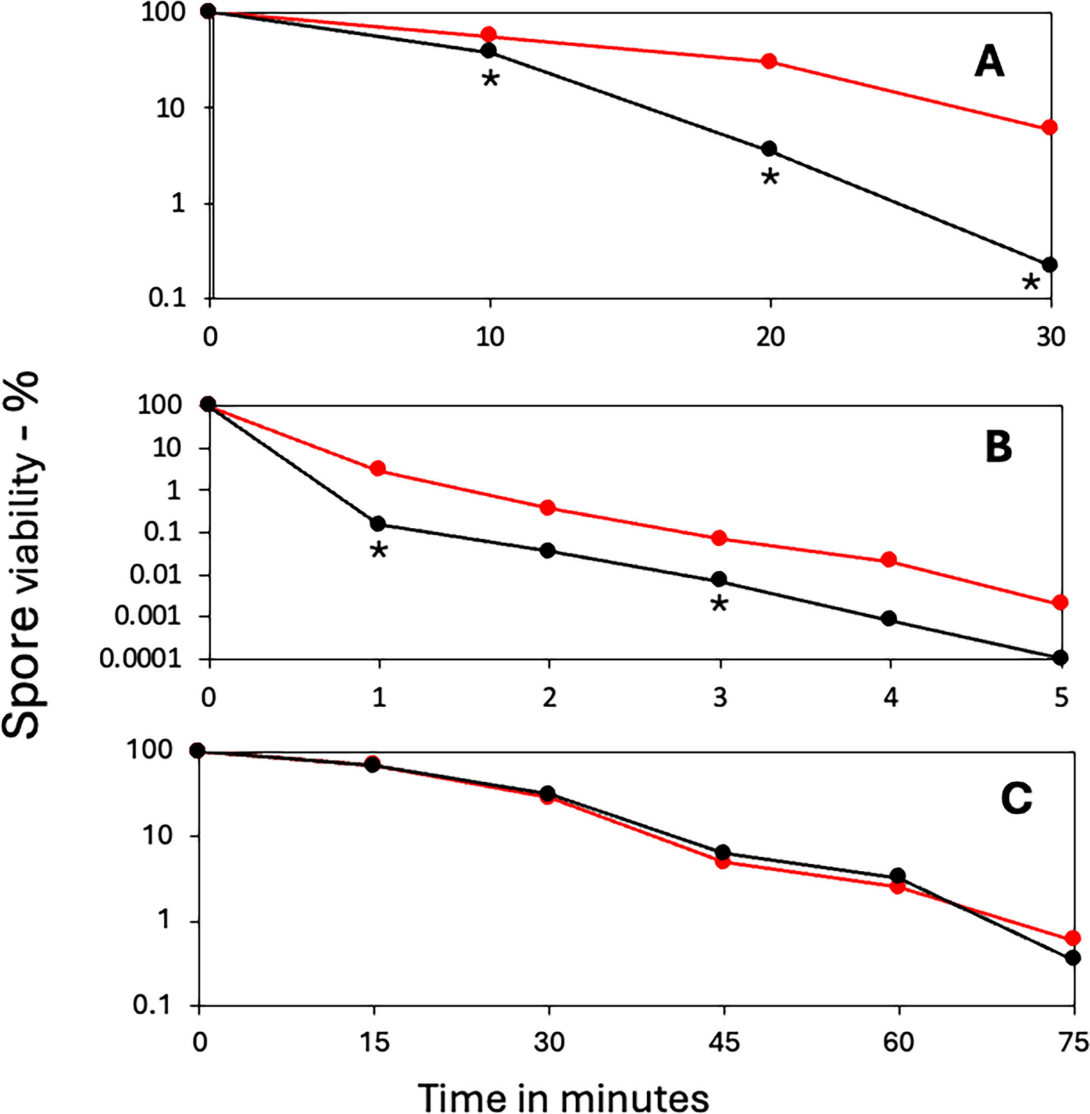
Resistance of spores of PY79 strains with or without sporulene to various agents. Spores of PY79 wt and its sporulene-less mutant, PS4567, were treated with (**A**) H_2_O_2_, (**B**) UV light, and (**C**) wet heat at 93°C, and spore viability was determined as described in Materials and Methods. All values are averages from duplicate determinations in at least two independent experiments. The symbols used are as follows: red, PY79, wt; and black, PS4567. Statistical significance was determined by unpaired *t*-tests comparing sporulene-less spores (PS4567) to wild-type PY79 spores at each time point. * indicates *P* < 0.05 for differences between PS4567 and PY79 wt; the absence of an asterisk indicates no significant differences (*P* > 0.05).

The absence of sporulene or a sporulene precursor was previously shown to decrease spore resistance to H_2_O_2_ (9, 10, 12), and this was confirmed for PS832 and PY79 wt strains (Fig. 2A and 3A). In addition, sporulene-less spores exhibited identical wet heat resistance to wt spores (Fig. 2C and 3C), while spore UV resistance was significantly lower in sporulene-less PS832 and PY79 spores (Fig. 2B and 3B). Importantly, removal of the antibiotic resistance marker had no effect on PS832 spore resistance to wet heat, UV, or hydrogen peroxide (Fig. 2B to D). Note that the experiments comparing the resistance of spores of strains with or without antibiotic markers were very important, as recent work (26) found that the presence of antibiotic markers and/or other foreign DNA in *Bacillus cereus* can lead to significant effects on spore proteomes, even if antibiotics are not present.

**Fig 4:**
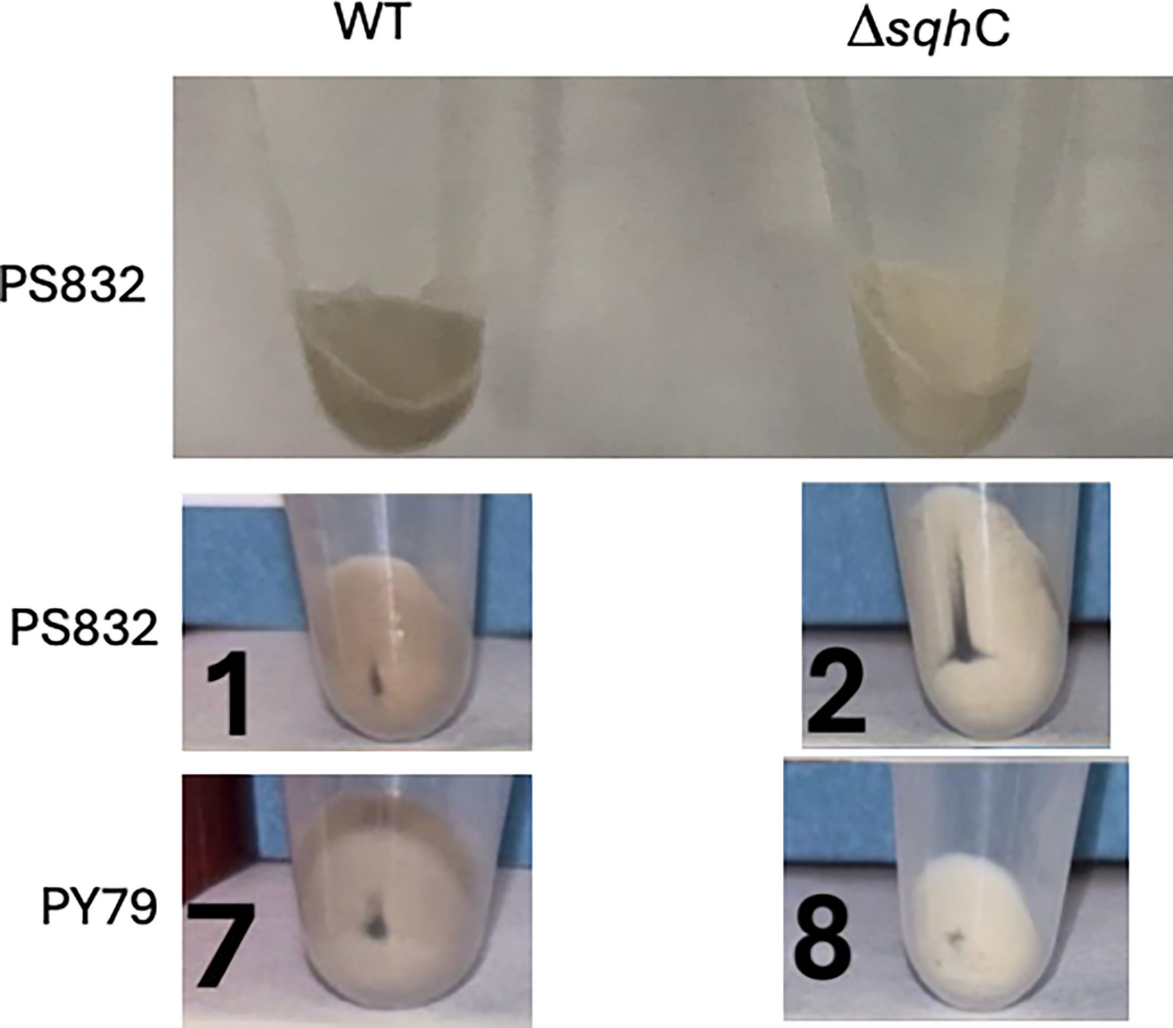
Photographs of ~2 mL of PS832 or PY79 spores with (wt) or without (Δ*sqhC*) sporulene at an OD_600_ of ~25. Spores were pelleted in 2 mL microcentrifuge tubes. Tubes were photographed. Spores were made on plates and purified as described in Materials and Methods and are (1) PS832 (wt), (2) PS4559 (PS832 Δ*sqhC*), (7) PY79 (wt), and (8) PS4567 (PY79 Δ*sqhC*). Note that the two PS832 spore preps were made about 1 year apart and used different batches of nutrient broth in the spore production plates, but the wt and Δ*sqhC* spores whose darkness was compared were made on plates with the same batch of nutrient broth.

It was also notable that while suspensions of spores with or without bacillithiol looked identical in terms of their color (data not shown), pellets of PS832 and PY79 spores lacking sporulene were whiter than pelleted wt spores (Fig. 4). Note that wt spore pellets’ levels of darkness varied somewhat between spore preps, likely due to differences in the batches of medium components used for different spore preps. However, sporulene-less spores prepared in parallel with wt spores were invariably whiter than the wt spores.

### The absence of bacillithiol or sporulene had effects on spore germination

**Fig 5:**
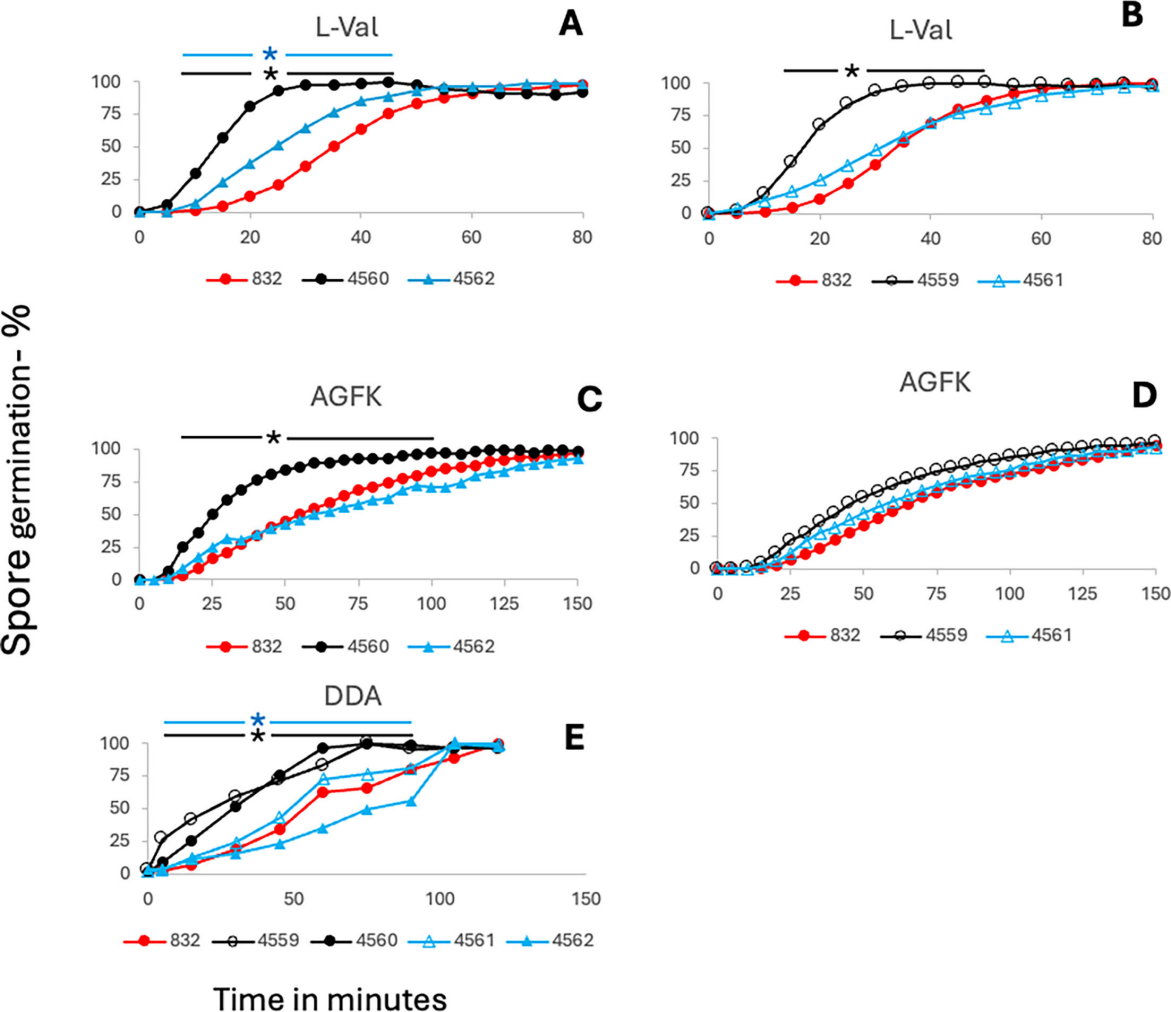
Germination with various germinants of PS832 wt spores and spores lacking bacillithiol or sporulene and plus or minus antibiotic markers. Spores were heat-activated and germinated with either (**A and B**) L-valine, (**C and D**) AGFK, or (**E**) without heat activation and germinated with dodecylamine. Spore germination was followed by measuring DPA release by its fluorescence with Tb^3+^ as described in Materials and Methods; symbols for strains are given below the *x* axes. Statistical significance was determined by one-way ANOVA followed by unpaired *t*-tests (**A–D**) or one-way ANOVA followed by pairwise *t*-tests (**E**). Black and blue asterisks indicate significant differences (*P* < 0.05) for specific pairwise comparisons at the indicated time points in regions shown by lines for the following comparisons: in panels A, B, C, and D between PS832 wt and PS4560 and PS4562 and between PS832 wt and PS4559 and PS4562; in panel E, between any pair among PS832 wt, PS4559, PS4560, PS4561, and PS4562; absence of an asterisk indicates no significant difference (*P* > 0.05).

**Fig 6:**
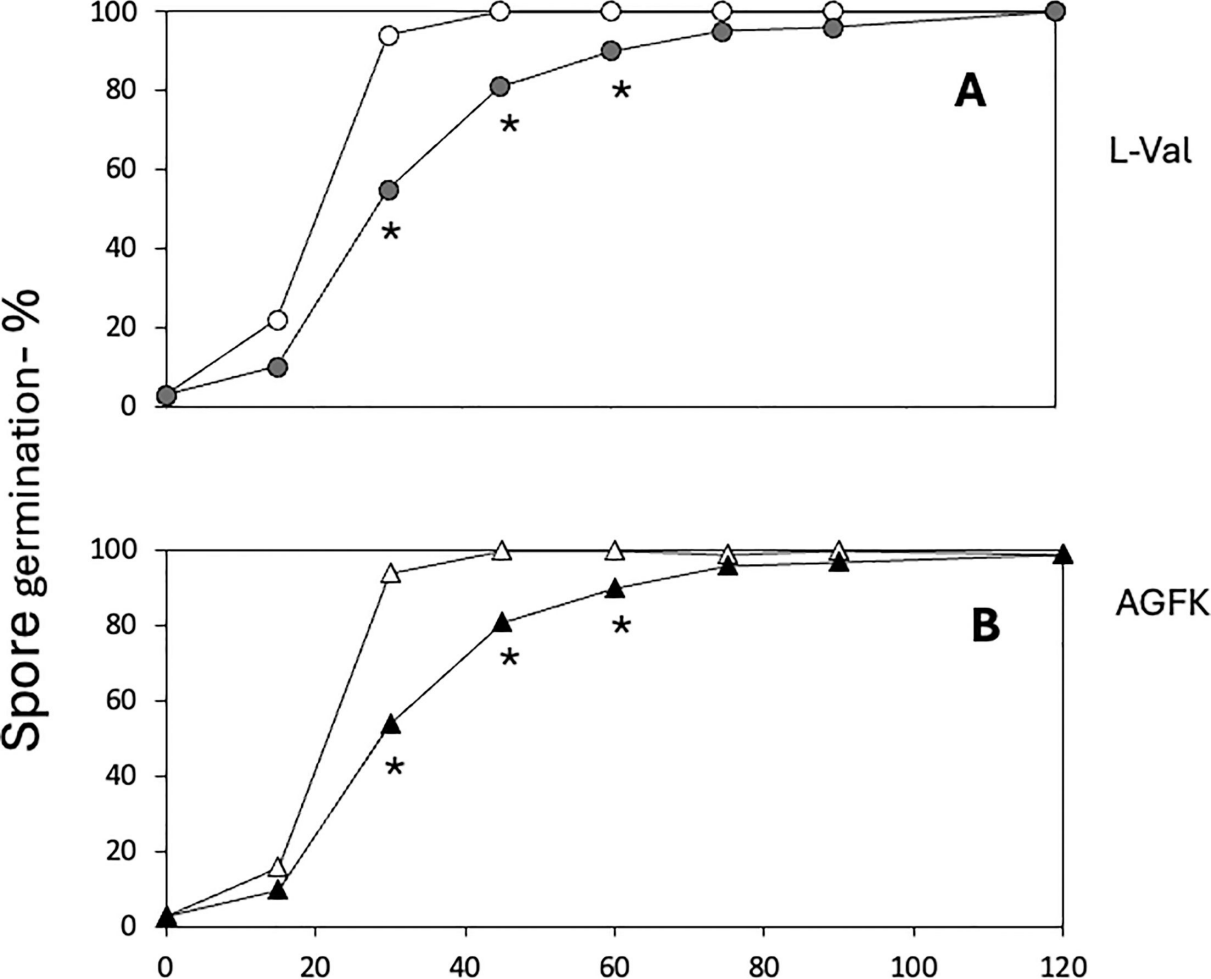
Germination of PSY79 spores with or without sporulene. Spores of strains were heat-activated and germinated with either (**A**) L-valine or (**B**) AGFK, and spore germination was followed by measuring DPA release by its fluorescence with Tb^3+^ as described in Materials and Methods. All values are averages of quadruplicate measurements. The symbols used are as follows: open symbols, PY79 wt; filled symbols, PS4597 (PY79 lacking sporulene). Statistical significance was determined by one-way ANOVA followed by pairwise unpaired *t*-tests comparing all possible pairs of the four strains at each time point. * indicates *P* < 0.05 for differences between PS4567 and PY79 wt; the absence of an asterisk indicates no significant differences (*P* > 0.05).

Rates of L-valine germination via the GerA GR were increased in PS832 spores lacking sporulene, with bacillithiol’s absence having a similar effect (Fig. 5A and B). An increase in the rate of AGFK germination via the GerB and GerK GRs was also seen with spores lacking *sqhC*, although the absence of bacillithiol had at most a minimal effect (Fig. 5C and D). In addition, spores of PS832 lacking sporulene or bacillithiol germinated faster with dodecylamine than did the respective wt spores (Fig. 5E). However, all effects of the lack of sporulene or bacillithiol on PS832 spore germination were eliminated if the antibiotic markers replacing the *sqhC* or *bshC* genes were also removed (Fig. 5A through D). In contrast to the faster L-valine and AGFK germination of PS832 spores lacking sporulene (Fig. 5B and D), the rates of L-valine and AGFK germination were reduced considerably in PY79 spores lacking sporulene (Fig. 6A and B), although effects of removal of the antibiotic marker were not tested.

### IM permeability and levels of core water in wt, sporulene-less, and bacillithiol-less spores

**Fig 7:**
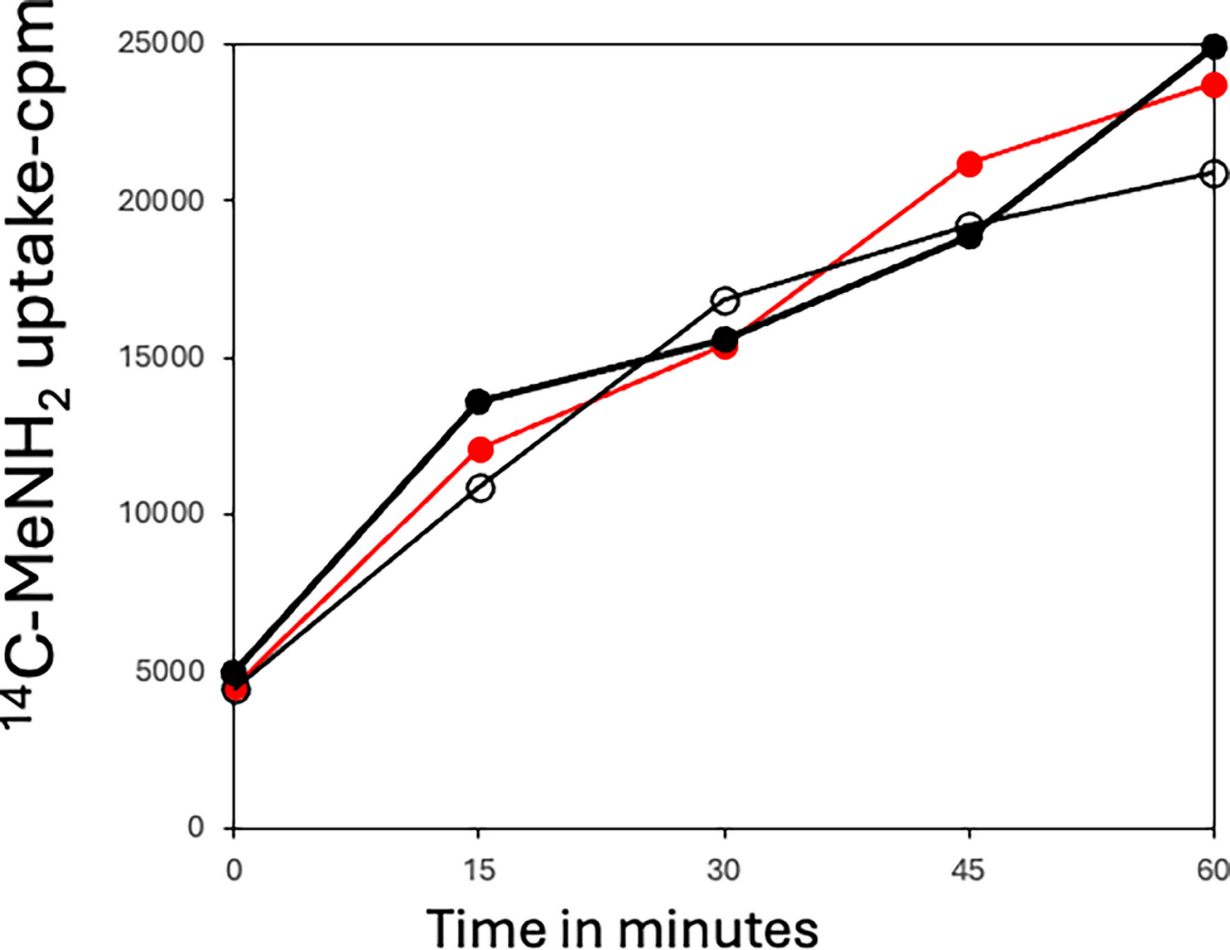
^14^C-Methylamine uptake by spores of various *B. subtilis* strains. The spores used are as follows red filled circle, PS832 (wt);, black open circle, PS4559 (no sporulene); and black filled circle, PS4560 (no bacillithiol). Measurements of ^14^C-methylamine uptake were averages of duplicate determinations, as described in Materials and Methods.

**Fig 8:**
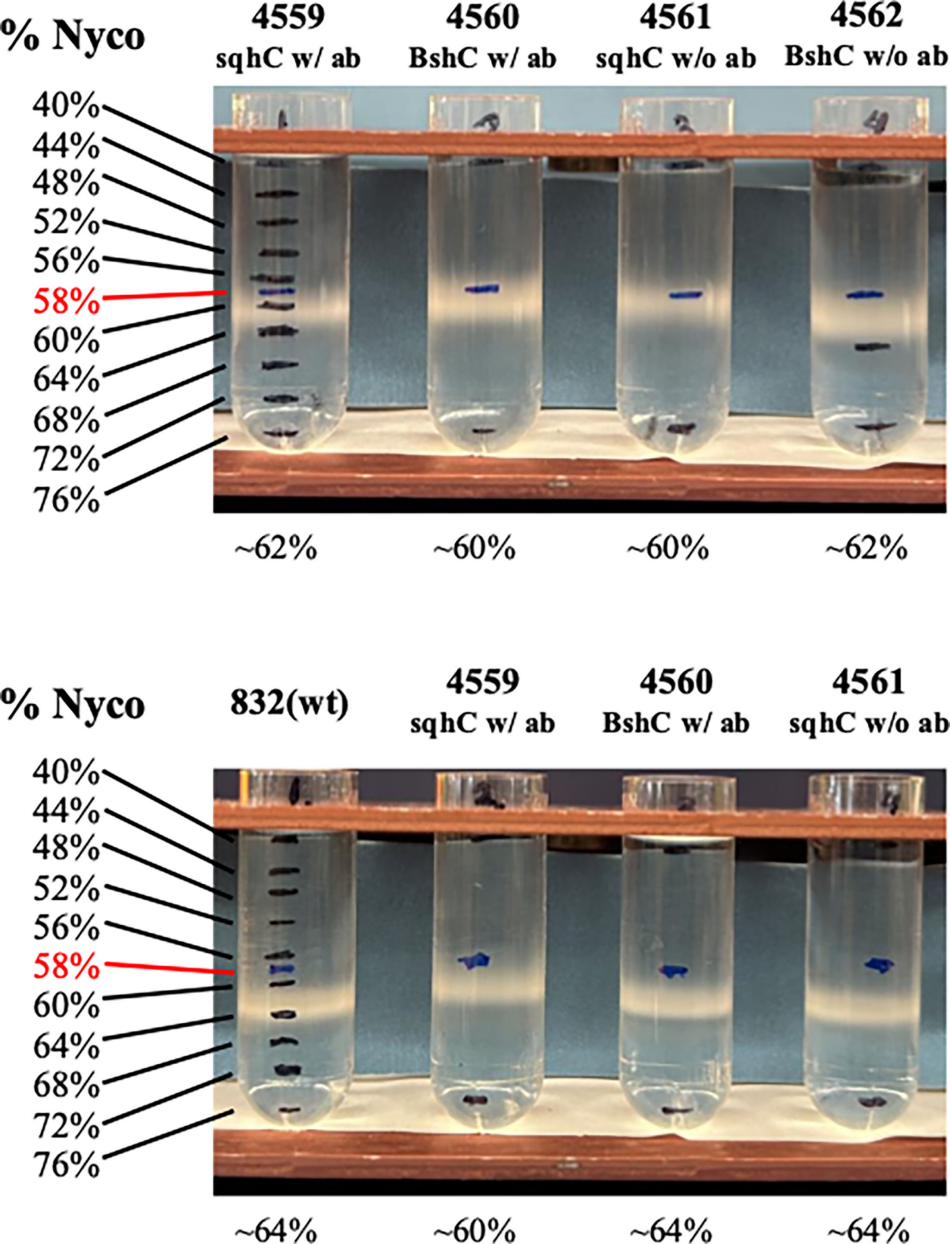
Measurements of core water content in spores of strains with and without sporulene, bacillithiol, and antibiotic markers. Purified spores of various strains (Table 1) were centrifuged to equilibrium on 40%–76% Nycodenz gradients as described in Materials and Methods, and the centrifuge tubes with the gradients and spores were photographed. Positions of various densities are shown by the black bands, with the red band being the midpoint. The positions of banding of the various spores are given below the centrifuge tubes. Note that strains given above the tubes are either with the antibiotic marker (with ab) in the gene shown or without it (w/o ab).

Since PS832 spores are known to have relatively low rates of permeability into the spore core (27, 28), spores ± sporulene or bacillithiol were also tested for the permeability of ^14^C-methylamine (27), and spores of all strains tested exhibited essentially identical rates of methylamine uptake (Fig. 7). Spores of these three strains were also prepared on plates containing 250 µM Laurdan, a dye that is incorporated into spores’ IM, and is responsive to IM fluidity, with larger generalized polarization (GP) values indicating a more rigid IM (7, 8, 22, 23) (Table 2). The core water contents of spores with and without sporulene or bacillithiol were also measured, and the differences between the wt and mutant spores were minimal (Fig. 8).

**TABLE 2:** GP values of laurdan labeled PS832 spores ± sporulene or bacillithiol and PY79 spores ± sporulene^a^

Spores examined	GP values	SD values	Number of spores examined
PS832 (wt)	−0.11	0.03	60
PS4559 (Δ*sqhC)*	−0.03	0.03	60
PS4560 (Δ*bshC*)	−0.13	0.03	60
PY79 (wt)	−0.12	0.02	60
PY79 (Δ*sqhC*)	−0.20	0.04	60

^a^ Laurdan-labeled spores were prepared and purified, and GP values were determined as described in Materials and Methods.

## DISCUSSION

The work reported in this manuscript provides new insight into two additional molecules that modulate *B. subtilis* spores’ germination and resistance (2). Notably, the pigmentation due to sporulene and its precursor curcumene greatly increased the spores’ UV resistance. Presumably, this is by absorbing radiation in the spores’ outer layers and protecting DNA in the spore core. Indeed, previous work has found that pigments such as carotenes in spores’ outer layers markedly increase spores’ UV resistance (29–31). The decreased spore hydrogen peroxide resistance due to sporulene was reported previously (9, 12) and is likely due to inactivation of this agent in spores’ outer layers, thus minimizing its permeation into the spore core, where it causes lethal damage. The absence of the major thiol, bacillithiol, in *B. subtilis* also appears important in spore wet heat resistance, although how this occurs is not clear. The other major thiol in dormant spores, Coenzyme A, is present in the spore core primarily in disulfide linkage to core proteins because of the core’s minimal, if any, supply of reducing agents such as NADH (32, 33). While how the absence of bacillithiol reduces spore wet heat resistance is not clear, it could be by disulfide formation protecting crucial protein thiol groups against damage accelerated at high temperatures.

The absence of sporulene or bacillithiol from PS832 spores also increased spore germination rates, but the absence of sporulene decreased PY79 spores’ germination, and these effects were significant. However, with PS832 spores, removal of the antibiotic marker that replaced the *sqhC* or *bshC* genes largely restored wild-type spore germination. Previous work has shown that the incorporation of antibiotic markers or other foreign DNA into *B. cereus* can cause dramatic changes in the resultant spores’ proteomes (26), although how this occurs is not clear. Perhaps this is also true, at least to some degree, in *B. subtilis,* and this might explain the effects of replacement of the *sqhC* and *bshC* genes by antibiotic markers on spore germination rates. This is certainly something that needs further study and to be kept in mind when making deletion/replacement mutations in *B. subtilis* going forward.

Spore germination proteins are largely in spores’ IM, and IM permeability and rigidity are very important in spore resistance to small molecules; IM fluidity is a crucial parameter in determining rates of spore germination, with faster germination associated with a more fluid IM (5–8, 19). The absence of sporulene or bacillithiol had no effects on PS832 spores’ IM permeability or core water content, but sporulene’s absence increased PS832 spores’ germination rates but decreased germination rates of PY79 spores. However, only slightly decreased IM fluidity is predicted in sporulene-less spores based on the GP values of the dye Laurdan in PS832 spores’ IM (22, 23 and Table 2). Slightly increased IM fluidity is predicted in sporulene-less spores based on the GP values in PY79 (Table 2). Ramana et al. (12) suggest that the lack of sporulenes led to spores with a fragile inner membrane, which made them susceptible to the loss of cultivability. Thus, even with a more fluid membrane, they could still germinate, but at a slower rate. Notably, molecules with similarities to sporulene, including its precursor curcumene and cholesterol, can all significantly modulate membrane properties (15, 23, 34–36), and thus, at least some effects of sporulene on spore IM properties seem likely.

Sporulene also had other significant effects on spore properties, as the increased UV resistance due to sporulene seems most likely due to increased spore pigmentation by sporulene and its precursor curcumene, and other classes of pigments also increase spores’ UV resistance (28–30). The resistance to hydrogen peroxide due to sporulene is also significant, as reported some years ago (9). This could be due to greatly decreasing spore IM permeability such that H_2_O_2_ does not easily enter the spore core, where it does its lethal damage. However, spores’ IM permeability to methylamine was unchanged in spores with or without sporulene or bacillithiol. Thus, it is more likely that sporulene, presumably in spores’ outer layer, the site of its precursor curcumene (14), is inactivated by its reaction with hydrogen peroxide. Indeed, hopanoids, molecules related to sporulene, have the capability to generate an oxygen barrier in some microbes, thus preventing oxygen access to O_2_-sensitive proteins (33–35). It is also notable that there is no change in spore core water content when sporulene is absent, and thus, this spore parameter affecting spore resistance is not altered. One unknown about sporulene in spores is exactly where they are. This is almost certainly in spores’ outer layer, which is where its precursor curcumene has been found (15). Given that relatives of sporulene have been shown to accumulate on microorganisms’ surfaces and provide strong protection against a variety of agents (34–36), perhaps future work is needed to localize sporulene in spores.

### Conclusion

The work in this manuscript identifies new roles that sporulene plays in spores’ germination and resistance, both inactivating toxic chemicals and UV light and altering spores’ IM properties and thus spores’ germination and resistance. This also appears to be the case for bacillithiol. Overall, this work adds two additional agents to the long list of effectors of *Bacillu*s spore resistance and germination. The insertion of antibiotic markers to remove genes for synthesis of bacillithiol or sporulene also led to some large effects on spore germination and formaldehyde resistance. This was unexpected. However, previous work (26) found that insertion of an antibiotic resistance marker into *B. cereus* resulted in significant changes to the resulting spores’ proteomes. Perhaps this is also true for antibiotic resistance markers in *B. subtilis*!

## Data Availability

Data will be made available on request.
